# Human papilloma virus E7 oncoprotein abrogates the p53-p21-DREAM pathway

**DOI:** 10.1038/s41598-017-02831-9

**Published:** 2017-06-01

**Authors:** Martin Fischer, Sigrid Uxa, Clara Stanko, Thomas M. Magin, Kurt Engeland

**Affiliations:** 1 Molecular Oncology, Medical School, University of Leipzig, Leipzig, Germany; 2 Institute of Biology and Translational Center for Regenerative Medicine, University of Leipzig, Leipzig, Germany

## Abstract

High risk human papilloma viruses cause several types of cancer. The HPV oncoproteins E6 and E7 are essential for oncogenic cell transformation. E6 mediates the degradation of the tumor suppressor p53, and E7 can form complexes with the retinoblastoma pRB tumor suppressor. Recently, it has been shown that HPV E7 can also interfere with the function of the DREAM transcriptional repressor complex. Disruption of DREAM-dependent transcriptional repression leads to untimely early expression of central cell cycle regulators. The p53-p21-DREAM pathway represents one important means of cell cycle checkpoint activation by p53. By activating this pathway, p53 can downregulate transcription of genes controlled by DREAM. Here, we present a genome-wide ranked list of genes deregulated by HPV E7 expression and relate it to datasets of cell cycle genes and DREAM targets. We find that DREAM targets are generally deregulated after E7 expression. Furthermore, our analysis shows that p53-dependent downregulation of DREAM targets is abrogated when HPV E7 is expressed. Thus, p53 checkpoint control is impaired by HPV E7 independently of E6. In summary, our analysis reveals that disruption of DREAM through the HPV E7 oncoprotein upregulates most, if not all, cell cycle genes and impairs p53’s control of cell cycle checkpoints.

## Introduction

High risk human papilloma viruses (HPV) are oncogenic DNA viruses that can cause cancer of the cervix uteri, oropharynx, penis, vagina, vulva and anus^1–4^. The primary transforming capacities of HPV stem from the E6 and E7 proteins. These two oncoproteins cooperate in silencing the anti-proliferative control of the cell. The best-known target of E7 is the cell cycle regulator pRB^5^. Direct binding of E7 to the retinoblastoma tumor suppressor protein pRB impairs its function^6–8^. Furthermore, association of E7 with pRB leads to an increase in p53 levels^9^. The tumor suppressor p53 can either trigger checkpoints causing cell cycle arrest or lead to the induction of apoptosis. In the context of HPV infection, the E6 oncoprotein initiates degradation of p53^10, 11^.

Moreover, the E7 oncoprotein has additional functions aside from targeting pRB^12, 13^. These include means to impair p53 function even in the absence of E6. Thus, HPV E7 is sufficient to block cell cycle checkpoint control by p53^14–18^. The cyclin-dependent kinase (CDK) inhibitor p21 (CDKN1A) is a central mediator of p53 checkpoint control^19, 20^, and its function can be impaired by E7^18, 21, 22^. This finding is particularly interesting given that p21 is required for downregulation of genes in response to p53^23, 24^.

Recently, another mechanism to impair p53 function that is independent of E6 was discovered. HPV E7 was found to disrupt the pRB-related transcriptional repressor complex DREAM (DP, RB-like, E2F4 and MuvB)^25–27^. The DREAM protein complex consists of E2F4, DP1 and p130/p107 in addition to RBBP4 and the LIN proteins LIN9, LIN37, LIN52 and LIN54 that form the MuvB core^28–30^. DREAM binds promoters through cell cycle-dependent elements (CDEs), cell cycle genes homology regions (CHRs), CHR-like elements (CLEs) and E2F sites^31–35^. In response to p53, DREAM is recruited to promoters of cell cycle genes, leading to their repression^36, 37^. While p53 itself is solely an activator of transcription, the p53-p21-DREAM pathway mediates indirect gene downregulation by p53^38, 39^. For example, *Polo-like kinase 4* is an important target of this pathway. The mitotic kinase PLK4 is repressed through the p53-p21-DREAM-CDE/CHR pathway^40^, and its p53-dependent repression can be abrogated by HPV E7^41^. Importantly, CDE/CHR elements are required for p53-dependent repression of *PLK4*, and the expression of HPV E7 impairs DREAM binding to the CDE/CHR elements in the *PLK4* promoter^41^. As genome-wide expression profiling datasets of E7-expressing IMR90 lung fibroblasts^42^ and NIKS keratinocytes^43^ became recently available, we asked whether targets of the p53-p21-DREAM pathway are generally deregulated by HPV E7 on a genome-wide level.

Here, we integrate these new data with earlier genome-wide datasets that were derived from comparing HPV-16/18-infected cervical tumor samples with normal tissue^44, 45^, from CaSki cells expressing HPV E2C, a potent transcriptional repressor of *E6* and *E7* ^46^, or from HeLa cells in which *E6* and *E7* were downregulated by RNAi^47^. Our analysis identifies genes that were observed as E7-regulated in most datasets, and we compared the results with lists of DREAM and pRB-E2F target genes^23^. We found that many DREAM targets are upregulated by E7 and that DREAM targets are the main class of genes deregulated by E7. Most importantly, p53-dependent downregulation of DREAM target genes is abrogated in HPV E7-expressing cells. In summary, our analysis provides a genome-wide high-confidence list of genes deregulated by HPV E7, most of which are DREAM targets. This study reveals the importance of E7-mediated DREAM disruption that interferes with p53-dependent gene downregulation. Thus, in HPV-infected cells, p53 function can be impaired by E7 independently of E6.

## Materials and Methods

### Computational analysis

A step-wise meta-analysis approach was employed to integrate multiple datasets^23^. This approach enables the integration of pre-analyzed datasets and does not require re-analysis of the raw data. Publicly available HPV E7 gene expression profiling datasets were curated^42–47^ and mapped to a collection of protein-coding genes^23^. Expression values of the analyzed genes were compiled and classified into downregulated (−1), upregulated (+1) and non-regulated genes (0).

Genes identified as significantly differentially regulated in HPV-16 E7 expressing NIKS cells were retrieved from Table 2 in Zhou *et al*.^43^. The pre-analyzed dataset of Rozenblatt-Rosen *et al*. from HPV-18 E7-expressing IMR90 cells was retrieved from the deposited Supplementary Table 19 in Rozenblatt-Rosen *et al*.^42^ and a gene was considered significantly differentially regulated if it passed the thresholds of adj. p-value ≤ 0.05 and absolute log2 (fold-change expression) ≥0.5. Genes identified as significantly differentially regulated in HPV-16/18 infected early stage cervical cancers compared to normal cervical epithelium were retrieved from the deposited Tables 2 and 3 in Santin *et al*.^45^. The pre-analyzed dataset from HeLa cells in which endogenous HPV-18 E6 and E7 expression was silenced by RNAi displays significantly differentially expressed genes and was retrieved from the Supplementary Table S1 in Kuner *et al*.^47^. Genes identified as significantly upregulated in HPV-16/18 infected primary cervical tumors compared to control cells were named “cervical cancer proliferation cluster” and were retrieved from Table 2 in Rosty *et al*.^44^. The pre-analyzed dataset from HPV E2C-expressing CaSki cells displays significantly differentially expressed genes and was retrieved from the Supplementary Table S1 in Pang *et al*.^46^. Of note, datasets by Rosty *et al*. and Pang *et al*. exclusively reported upregulated genes.

It is generally agreed that gene expression data from different experimental platforms are not directly comparable, and thus we used the stepwise meta-analysis approach instead that ranks genes by the number of datasets that find them significantly differentially regulated. Given that raw data were not re-analyzed, the approach does not include data points that were below the thresholds set in the individual studies. Differences in unprocessed data acquisition between several studies may reduce reproducibility, yet it minimizes the bias that would be introduced by using one particular analysis approach for all datasets. Following the stepwise meta-analysis approach^23^, genes were ranked by the number of datasets finding the gene to be significantly upregulated minus the number of datasets that find the gene to be downregulated (Supplementary Table S1).

### Cell culture and drug treatment

HCT116 cells were grown in Dulbecco’s modified Eagle’s medium (DMEM; Lonza, Basel, Switzerland) supplemented with 10% fetal calf serum (FCS) (Biochrom, Berlin, Germany) and penicillin/streptomycin and maintained at 37 °C and 10% CO_2_. Stably transfected HCT116 cells were generated by transfection with pCMV-HPV16-E7 wt (kindly provided by Karl Münger^48^), and selection with G418/Geneticin (PAA Laboratories, Pasching, Austria) at a concentration of 0.5 mg/ml^41^. Wild-type mouse keratinocytes were isolated from C57BI6 mouse embryos as described previously^49^. Cells were grown on plates coated with collagen (Invitrogen, Darmstadt, Germany) and maintained at 10% CO_2_ and 32 °C in DMEM/Ham’s F12 (3.5:1.1) (PAN Biotech, Aidenbach, Germany). Cells were treated with doxorubicin (0.2 μg/ml; Medac, Wedel, Germany) or Nutlin-3a (10 μM; Cayman Chemicals, Ann Arbor, MI, USA) for 24 h. For cell sorting of transiently transfected wild-type mouse keratinocytes, pEGFP plasmid (Clontech, Mountain View, CA, USA) was co-transfected with pCMV-HPV16-E7 wt plasmid at a 1:3 ratio using GeneJuice (Merck, Darmstadt, Germany). Fluorescence-activated cell sorting was carried out on a FACS Aria SORP instrument (Becton Dickinson Biosciences, Franklin Lakes, NJ, USA).

### RNA extraction, reverse transcription and semi-quantitative real-time PCR

Total cellular RNA was isolated using TRIzol reagent (Invitrogen, Carlsbad, CA, USA) following the manufacturer’s protocol. One-step reverse transcription and quantitative real-time PCR were performed with an ABI 7300 Real-Time PCR System (Applied Biosystems, Forster City, CA, USA) using QuantiTect SYBRGreen PCR Kit (Qiagen, Hilden, Germany) as described previously^41^. Primer sequences have been published previously^34, 40, 41, 50, 51^.

### Sodium dodecyl sulphate-polyacrylamide gel electrophoresis and immunoblot

Sodium dodecyl sulphate-polyacrylamide gel electrophoresis and western blot were performed following standard protocols^52^. The following antibodies were used: E2F1 (sc-193, Santa Cruz Biotechnology, Santa Cruz, CA, 1:500 dilution), KIF23 (sc-136473, Santa Cruz Biotechnology, 1:200), CDC25C (sc-327, Santa Cruz Biotechnology, 1:1000), B-MYB (LX015.1, kindly provided by Roger Watson^53^, hybridoma media 1:5) and ß-actin (A5441, Sigma-Aldrich, Munich, Germany, 1:5000).

## Results

### The HPV E7 oncoprotein deregulates cell cycle genes targeted by the DREAM complex

Two recently published datasets identified genes deregulated upon expression of the HPV E7 oncoprotein on a genome-wide basis^42, 43^. Combined, those two datasets identified 753 genes deregulated by E7, including 453 upregulated and 300 downregulated genes (Table S1). A fraction of these genes was identified in both datasets, 66 upregulated and 2 downregulated genes (Table 1). The small number of genes downregulated upon E7 expression indicates that expression of E7 primarily causes gene upregulation. When comparing the overlap of the 66 upregulated genes with recently published lists of cell cycle genes and targets of the DREAM, MMB-FOXM1 and pRB-E2F complexes^23^, it becomes evident that most E7-upregulated genes are cell cycle genes and targets of the DREAM complex (Table 1).

**Table 1:** HPV E7 deregulates DREAM target genes.

Gene Symbol	Cell cycle gene	DREAM target	Gene Symbol	Cell cycle gene	DREAM target
*APOBEC3B*	G2/M	✓	*MCM2*	G1/S	✓
*ASF1B*	G1/S	✓	*MCM3*	G1/S	✓
*ATAD2*	G1/S	✓	*MCM4*	G1/S	✓
*ATAD5*	UNKN	✓	*MCM5*	G1/S	✓
*BRCA1*	G1/S	✓	*MCM6*	G1/S	✓
*BRCA2*	G1/S	✓	*MCM7*	UNKN	✓
*BRIP1*	G1/S	✓	*MMS22L*	G1/S	✓
*CCNE2*	G1/S	×	*MSH2*	G1/S	✓
*CDC25A*	G1/S	✓	*MSH6*	G1/S	✓
*CDC45*	G1/S	✓	*MTBP*	G1/S	✓
*CDC6*	G1/S	✓	*MYBL2*	G1/S	✓
*CDC7*	G1/S	✓	*NCAPG2*	G1/S	✓
*CDK2*	G1/S	✓	*NUSAP1*	G2/M	✓
*CDKN2A*	×	×	*ORC1*	G1/S	✓
*CENPK*	G1/S	✓	*PCNA*	G1/S	✓
*CENPQ*	G1/S	✓	*POLA1*	G1/S	✓
*CENPU*	G1/S	✓	*POLD3*	G1/S	✓
*CHAF1A*	G1/S	✓	*POLE*	G1/S	✓
*CHAF1B*	G1/S	×	*PRIM1*	G1/S	✓
*DONSON*	G1/S	×	*RAD51AP1*	G1/S	✓
*DSN1*	G1/S	✓	*RBL1*	G1/S	✓
*DTL*	G1/S	✓	*RFC3*	G1/S	✓
*E2F1*	G1/S	✓	*RFC5*	G1/S	✓
*EMP2*	G1/S	×	*RRM2*	G1/S	✓
*FAM111B*	G1/S	✓	*SASS6*	UNKN	✓
*FANCI*	G1/S	✓	*STIL*	G2/M	✓
*FANCL*	G1/S	✓	*TICRR*	G2/M	✓
*FIGNL1*	G1/S	✓	*TMPO*	G2/M	✓
*GINS1*	G1/S	✓	*UHRF1*	G1/S	✓
*GINS2*	G1/S	✓	*WDHD1*	G1/S	✓
*GMNN*	G1/S	✓	*WDR76*	G1/S	✓
*HELLS*	G1/S	✓	*ZWINT*	G1/S	✓
*KNTC1*	G1/S	✓	*AMIGO2*	G1/S	×
*MASTL*	G1/S	✓	*RHOB*	×	×

68 genes overlap in two datasets of genes deregulated upon HPV E7 expression^42, 43^. Two genes described in both datasets as downregulated are underlined. Annotation of genes as DREAM targets or cell cycle genes, including the cell cycle phase of peak expression, were extracted from Fischer *et al*.^23^. UNKN, timing of peak expression of the cell cycle gene is unknown; X, cell cycle-dependent expression was not reported in the datasets.

Next, we integrated additional datasets that identified genes that are deregulated by HPV E6 and E7^44–47^, employing tools and data from a recent meta-analysis^23^. By combining six datasets and using stringent thresholds, this approach yields reliable target identification. In total, these six datasets identified 1,783 genes as deregulated by HPV E7 (Table S1). No gene was identified in all datasets as downregulated by E7, further supporting the notion that E7 expression primarily results in target gene induction. Fourteen genes were identified as upregulated by E7 in at least five of the six datasets. Remarkably, all of these genes are cell cycle genes and DREAM targets. Furthermore, when looking at the 49 genes identified in at least four of the six datasets, 34 are cell cycle genes and DREAM targets (Table 2). Only one gene from this group, *CDKN2A* (*p16*), is not a DREAM target. *CDKN2A* was previously reported to be upregulated by HPV E7 through a different mechanism, namely epigenetic derepression^54^.

**Table 2:** HPV E7-deregulated genes with an identification-overlap of at least four out of six datasets.

Gene Symbol	Identified as upregulated by E7 in No. of datasets	Cell cycle gene	DREAM target	MMB-FOXM1 target	pRB-E2F target
*MCM2*	6	G1/S	✓	×	✓
*ZWINT*	6	G1/S	✓	×	×
*APOBEC3B*	5	G2/M	✓	×	×
*CDC6*	5	G1/S	✓	×	✓
*KIF2C*	5	G2/M	✓	✓	×
*LMNB1*	5	G2/M	✓	✓	✓
*MCM4*	5	G1/S	✓	×	✓
*MYBL2*	5	G1/S	✓	×	✓
*NUSAP1*	5	G2/M	✓	✓	✓
*PRC1*	5	G2/M	✓	✓	×
*RRM2*	5	G1/S	✓	✓	✓
*SMC4*	5	G2/M	✓	✓	✓
*STIL*	5	G2/M	✓	✓	✓
*TOP2A*	5	G2/M	✓	✓	×
*ASF1B*	4	G1/S	✓	✓	✓
*ASPM*	4	G2/M	✓	✓	×
*ATAD2*	4	G1/S	✓	✓	✓
*BIRC5*	4	G2/M	✓	✓	×
*BRCA1*	4	G1/S	✓	×	✓
*CCNA2*	4	G2/M	✓	✓	×
*CCNB1*	4	G2/M	✓	✓	×
*CCNB2*	4	G2/M	✓	✓	×
*CDC20*	4	G2/M	✓	✓	×
*CDC25C*	4	G2/M	✓	✓	×
*CDC45*	4	G1/S	✓	×	✓
*CDKN2A*	4	×	×	×	×
*CENPF*	4	G2/M	✓	✓	×
*DTL*	4	G1/S	✓	✓	✓
*E2F1*	4	G1/S	✓	×	✓
*FANCI*	4	G1/S	✓	×	✓
*FKBP5*	4	UNKN	✓	×	×
*FOXM1*	4	G2/M	✓	×	×
*GINS2*	4	G1/S	✓	×	✓
*KIF20A*	4	G2/M	✓	✓	×
*KIF23*	4	G2/M	✓	✓	×
*MELK*	4	G2/M	✓	×	×
*NCAPG2*	4	G1/S	✓	×	✓
*NEK2*	4	G2/M	✓	✓	×
*POLQ*	4	G2/M	✓	×	×
*PRIM1*	4	G1/S	✓	×	✓
*PTTG1*	4	G2/M	✓	✓	×
*RAD51AP1*	4	G1/S	✓	×	✓
*RFC3*	4	G1/S	✓	×	✓
*SPAG5*	4	G2/M	✓	✓	×
*TMPO*	4	G2/M	✓	×	✓
*TRIP13*	4	G2/M	✓	×	×
*TTK*	4	G2/M	✓	✓	×
*WDHD1*	4	G1/S	✓	×	✓
*WDR76*	4	G1/S	✓	×	✓

49 genes were identified in at least 4 of the 6 datasets as being deregulated by HPV E7 (compiled from Table S1). Annotation of cell cycle genes, including the phase of peak expression and DREAM, MMB-FOXM1 or pRB-E2F targets were extracted from Fischer *et al*.^23^. UNKN, timing of peak expression of the cell cycle gene is unknown; X, cell cycle-dependent expression was not reported in the datasets.

To be considered a high confidence HPV E7-deregulated gene, we employed a threshold of at least three datasets that identify the gene as upregulated by E7. Remarkably, 139 of 141 genes (98.6%) that passed these criteria are predicted cell cycle genes, and 134 (95.0%) are DREAM targets (Tables 2 and 3). The cell cycle genes represent genes with peak expression during G_1_/S or G_2_/M phases. Although pRB is the best known target protein of E7, only 87 (61.7%) of the high confidence E7-deregulated genes are predicted pRB-E2F targets. It is important to note that pRB-E2F targets largely represent the G_1_/S subgroup of DREAM-targeted cell cycle genes^23, 34^. The finding that most HPV E7-deregulated genes are DREAM targets is consistent with the previous finding that disruption of the DREAM complex is critical to prevent cell cycle arrest in ﻿HPV-i﻿nfec﻿ted c﻿ells^25^. Together, our findings indicate that DREAM target genes are generally deregulated by HPV E7 expression.

**Table 3:** Genes upregulated after HPV E7 expression with an identification-overlap of three in six datasets.

Gene Symbol	Identified as upregulated by E7 in No. of datasets	Cell cycle gene	DREAM target	MMB-FOXM1 target	pRB-E2F target	Gene Symbol	Identified as upregulated by E7 in No. of datasets	Cell cycle gene	DREAM target	MMB-FOXM1 target	pRB-E2F target
*ANLN*	3	G2/M	✓	✓	✓	*KNTC1*	3	G1/S	✓	×	✓
*ANP32E*	3	G2/M	✓	✓	✓	*MAD2L1*	3	G2/M	✓	✓	✓
*ATAD5*	3	UNKN	✓	✓	✓	*MASTL*	3	G1/S	✓	×	×
*AURKA*	3	G2/M	✓	✓	✓	*MCM10*	3	G1/S	✓	×	✓
*BRCA2*	3	G1/S	✓	×	✓	*MCM3*	3	G1/S	✓	×	✓
*BRIP1*	3	G1/S	✓	×	✓	*MCM5*	3	G1/S	✓	×	✓
*BUB1*	3	G2/M	✓	✓	×	*MCM6*	3	G1/S	✓	×	✓
*BUB1B*	3	G2/M	✓	✓	✓	*MCM7*	3	UNKN	✓	×	✓
*CCNE2*	3	G1/S	×	×	✓	*MKI67*	3	G2/M	✓	✓	×
*CCNF*	3	G2/M	✓	✓	×	*MMS22L*	3	G1/S	✓	×	✓
*CDC7*	3	G1/S	✓	×	✓	*MSH2*	3	G1/S	✓	×	✓
*CDCA3*	3	G2/M	✓	✓	×	*MSH6*	3	G1/S	✓	×	✓
*CENPA*	3	G2/M	✓	✓	×	*MTBP*	3	G1/S	✓	×	✓
*CENPE*	3	G2/M	✓	✓	×	*MTHFD1*	3	G1/S	✓	×	✓
*CENPK*	3	G1/S	✓	×	✓	*MYBL1*	3	UNKN	✓	×	×
*CENPN*	3	UNKN	✓	✓	✓	*NASP*	3	G1/S	✓	×	✓
*CENPQ*	3	G1/S	✓	×	✓	*NCAPG*	3	G2/M	✓	✓	✓
*CENPU*	3	G1/S	✓	×	✓	*NCAPH*	3	G2/M	✓	✓	×
*CHAF1A*	3	G1/S	✓	×	✓	*NDC1*	3	G2/M	✓	✓	×
*CHAF1B*	3	G1/S	×	×	✓	*NEMP1*	3	G1/S	✓	×	✓
*CKS1B*	3	G2/M	✓	✓	×	*OIP5*	3	G2/M	✓	✓	✓
*DDIAS*	3	G1/S	✓	×	×	*ORC1*	3	G1/S	✓	×	✓
*DHFR*	3	G1/S	✓	x	×	*PARP1*	3	G1/S	×	×	✓
*DLGAP5*	3	G2/M	✓	✓	×	*PBK*	3	G2/M	✓	×	×
*DNA2*	3	G1/S	✓	×	✓	*PCNA*	3	G1/S	✓	×	✓
*DONSON*	3	G1/S	×	×	✓	*PKMYT1*	3	G1/S	✓	×	×
*DSN1*	3	G1/S	✓	×	✓	*PLK1*	3	G2/M	✓	✓	×
*EMP2*	3	G1/S	×	×	✓	*POLA1*	3	G1/S	✓	×	✓
*EXO1*	3	G1/S	✓	×	✓	*POLA2*	3	G1/S	✓	×	✓
*EZH2*	3	G1/S	✓	✓	✓	*POLD1*	3	G1/S	✓	×	✓
*FAM111B*	3	G1/S	✓	×	✓	*POLD3*	3	G1/S	✓	×	✓
*FEN1*	3	G1/S	✓	×	✓	*POLE*	3	G1/S	✓	✓	✓
*FIGNL1*	3	G1/S	✓	×	×	*RACGAP1*	3	G2/M	✓	✓	×
*GINS1*	3	G1/S	✓	×	✓	*RBL1*	3	G1/S	✓	×	✓
*GMNN*	3	G1/S	✓	×	✓	*RFC4*	3	G1/S	✓	×	✓
*GTSE1*	3	G2/M	✓	✓	×	*RFC5*	3	G1/S	✓	×	✓
*H2AFZ*	3	UNKN	✓	✓	✓	*RMI1*	3	G1/S	✓	×	✓
*HAT1*	3	UNKN	✓	×	✓	*RNASEH2A*	3	G1/S	✓	×	✓
*HELLS*	3	G1/S	✓	×	✓	*SASS6*	3	UNKN	✓	×	×
*HMMR*	3	G2/M	✓	✓	×	*SMC2*	3	UNKN	✓	×	×
*ITGB3BP*	3	UNKN	✓	×	✓	*TICRR*	3	G2/M	✓	✓	✓
*KIAA0101*	3	G1/S	✓	×	✓	*TIMELESS*	3	UNKN	✓	×	✓
*KIF11*	3	G2/M	✓	✓	×	*TPX2*	3	G2/M	✓	✓	×
*KIF15*	3	G2/M	✓	✓	✓	*TYMS*	3	×	×	×	×
*KIF20B*	3	G2/M	✓	✓	×	*UBE2C*	3	G2/M	✓	✓	×
*KIF4A*	3	G2/M	✓	✓	×	*UHRF1*	3	G1/S	✓	×	✓

92 genes were identified in 3 of the 6 datasets as being deregulated by HPV E7 (extracted from Table S1). Information whether the gene is a cell cycle gene, including the phase of peak expression, and whether it is a DREAM, MMB-FOXM1 or pRB-E2F target were extracted from Fischer *et al*.^23^. UNKN, timing of peak expression of the cell cycle gene is unknown; X, cell cycle-dependent expression was not reported in the datasets.

### High risk HPV E7 abrogates p53-p21-DREAM-mediated repression of cell cycle genes

**Figure 1:**
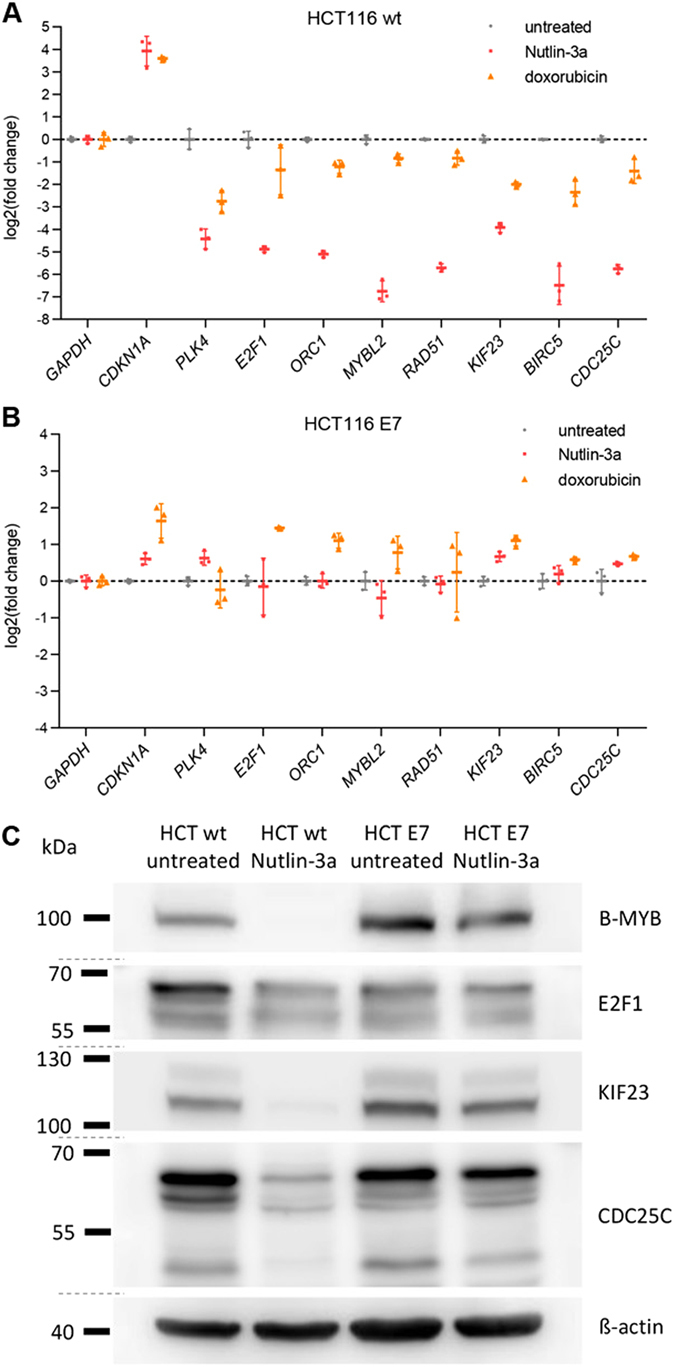
HPV E7 abrogates p53-mediated downregulation of DREAM target genes. (**A**) HCT116 wild-type and (**B**) HCT116 HPV E7-expressing cells were treated with Nutlin-3a or doxorubicin for 24 h. Untreated cells served as control. Semiquantitative RT-PCR was performed. The log 2-fold change of mRNA expression of treated compared to untreated cells is displayed. *GAPDH* served as a negative control for the p53 response, while *p21 (CDKN1A)* and *PLK4* were tested as positive controls. (**C**) HCT116 wild-type and E7-expressing cells were treated with Nutlin-3a for 24 h or left untreated. Protein levels were analyzed through immunoblotting and ß-actin levels served as loading control. Cropped blot images are displayed; full images are included in Supplementary Figure S1.

**Figure 2:**
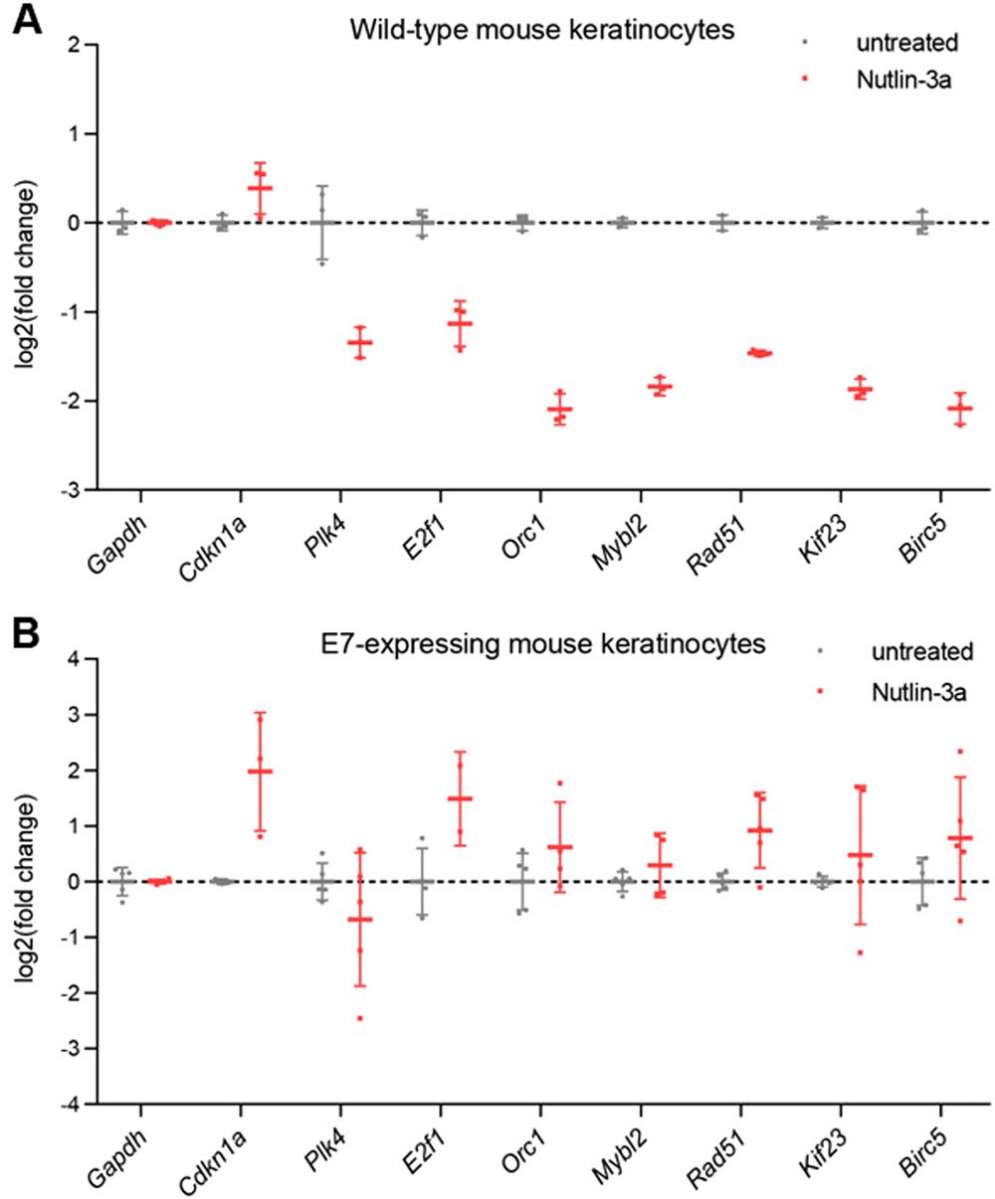
HPV E7 abrogates p53-mediated downregulation of DREAM target genes in wild-type keratinocytes. (**A**) Wild-type mouse keratinocytes were treated with Nutlin-3a for 24 h or left untreated. (**B**) Mouse keratinocytes were co-transfected with plasmids expressing HPV E7 and EGFP and treated with Nutlin-3a for 24 h or left untreated. Cells were sorted for green fluorescence followed by mRNA preparation. Relative mRNA expression was quantified by real-time RT-PCR and normalized to *GAPDH* RNA levels. The log2-fold change of mRNA expression is displayed for treated compared to untreated cells.

Given that p53-p21-dependent downregulation of the DREAM target gene *PLK4* was disturbed by HPV E7^41^, we asked whether disruption of the p53-p21-DREAM pathway was a general phenomenon upon HPV E7 expression. The p53-p21-DREAM pathway is best characterized in the HCT116 colon carcinoma cell line^37, 40^, and thus, we employed HCT116 cells stably transfected with HPV-16 E7 expression plasmids^41^. We treated wild-type and HPV E7-expressing HCT116 cells with p53-stabilizing Nutlin-3a or the DNA intercalator doxorubicin and compared changes in mRNA levels to untreated control cells (Fig. 1). Consistent with earlier findings, the mRNA levels of the well-established DREAM target genes *B-MYB* (*MYBL2*)^40^, *E2F1* ^23^, *CDC25C* ^51^, *Survivin* (*BIRC5*)^51^, *KIF23* ^50^, *ORC1* ^34^ and *RAD51* ^34^ were downregulated in HCT116 wild-type cells treated with Nutlin-3a or doxorubicin compared to untreated cells (Fig. 1A). Most importantly, downregulation of these genes was abrogated upon HPV E7 expression (Fig. 1B). With B-MYB (MYBL2), E2F1, KIF23 and CDC25C serving as examples, western blot analyses showed that protein levels followed decreased mRNA levels. Nutlin-3a treatment led to reduced B-MYB, E2F1, KIF23 and CDC25C protein levels in HCT116 wild-type cells but not in E7-expression HCT116 cells (Fig. 1C). In contrast to the abrogated repression of cell cycle genes, *p21* (*CDKN1A*) was still induced in response to p53 activation even when HPV E7 is present (Fig. 1B). Notably, HCT116 cells that express HPV E7 displayed an increased expression of DREAM target genes upon treatment with doxorubicin, but not in the presence of Nutlin-3a, when compared to untreated cells. Doxorubicin can induce G_1_/S and G_2_/M cell cycle arrest, while Nutlin-3a mainly induces G_1_/S arrest. A doxorubicin-induced increase in G_2_/M cell cycle population leads to increased mRNA levels of late cell cycle genes when the p53 pathway is not active or blocked, which has been observed previously^41, 50, 51^. To explore whether findings from HCT116 cancer cells are also observed in primary cells, we tested for mRNA expression changes following Nutlin-3a treatment in wild-type mouse keratinocytes compared to cells that were expressing HPV E7. Similar to the results from HCT116 cells, wild-type but not E7-expressing mouse keratinocytes displayed decreased mRNA levels of DREAM target genes upon Nutlin-3a treatment. Induction of *Cdkn1a* (*p21*), however, was not impaired by E7 expression (Fig. 2).

## Discussion

**Figure 3:**
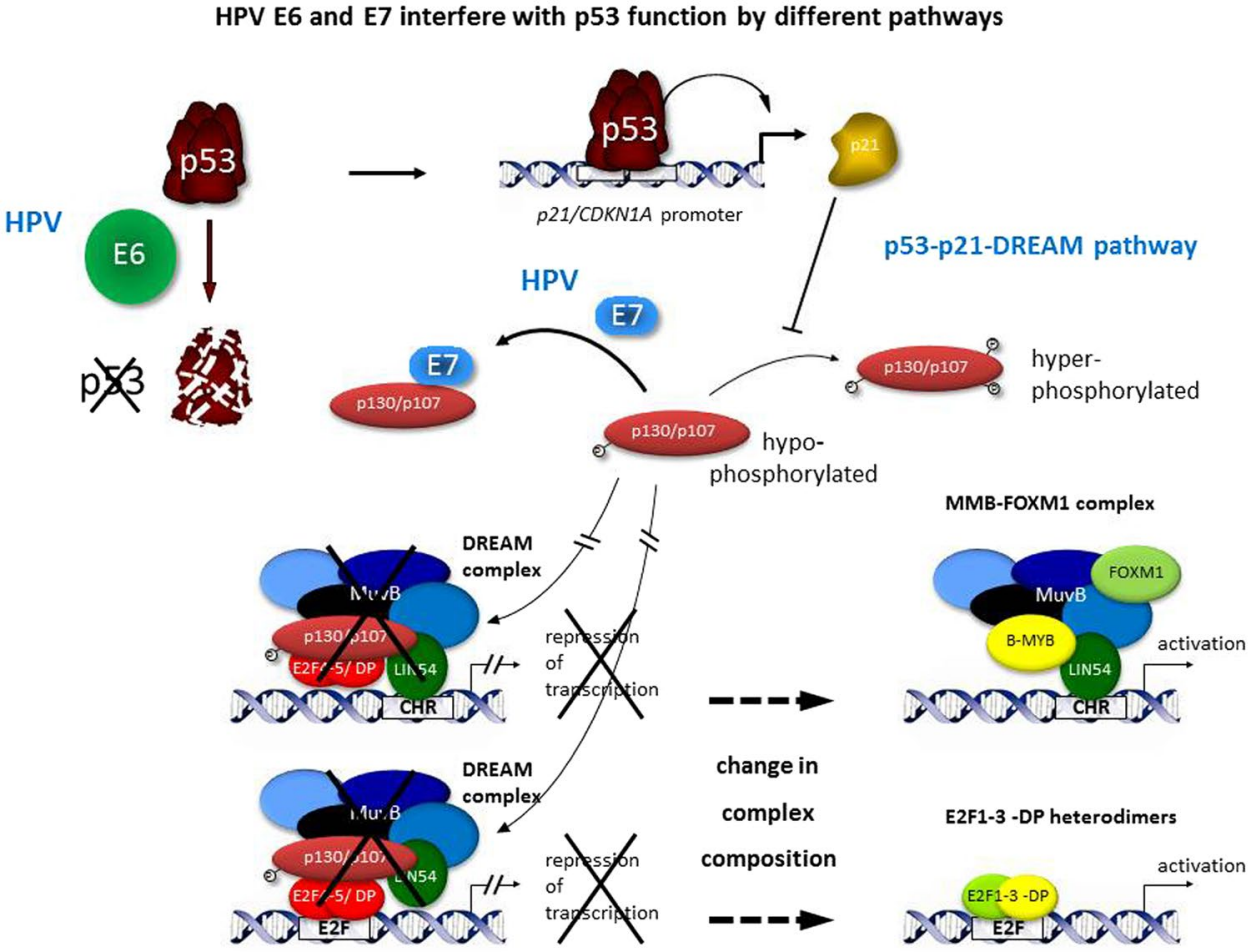
Both HPV E6 and E7 interfere with p53 function. HPV E6-mediated degradation of p53 is well established. By an independent mechanism, the HPV E7 oncoprotein interferes with the p53-p21-DREAM pathway. Interference is caused by the abrogation of indirect p53-dependent transcriptional repression of many genes required for cell cycle progression. E7 sequesters hypo-phosphorylated p130 and p107 proteins, thereby preventing them from forming the DREAM transcriptional repressor complex. In general, DREAM can bind to four combinations of promoter elements: CHR sites, E2F sites, CDE/CHR or E2F/CLE tandem elements^34^. For clarity only genes with CHR or E2F sites are depicted as examples. When p130/p107 pocket proteins are sequestered and not available for DREAM repressor formation, protein complexes on E2F or CHR sites change their composition from repressor to activator complexes. CHR elements then bind MMB-FOXM1 and E2F sites bind activating E2F1-3-DP complexes, respectively. In conclusion, sequestration of p130 and p107 by HPV E7 abrogates p53-dependent repression of cell cycle genes and thus impairs cell cycle checkpoint control by p53.

A cell uses several mechanisms to control proliferation. Hypo-phosphorylated forms of the pRB tumor suppressor block E2F-mediated induction of cell cycle genes required for the G_1_/S transition^55^. In addition, activation of proliferation in cells with serious defects in replication leads to DNA damage and causes stabilization of p53, which triggers cell cycle arrest or apoptosis^56^. By employing E6 and E7 oncoproteins, human papilloma viruses have evolved two strategies to intercept the host’s control of proliferation and response to infection. HPV E6 causes destruction of p53^10, 11^, and E7 forms a complex with pRB, thereby interfering with pRB’s ability to form complexes with E2F transcription factors^6–8^. Recently, a third mechanism based on E7 preventing DREAM complex formation was discovered^25, 26, 41^. Here, we analyzed E7-mediated gene dysregulation using genome-wide data analysis and expression profiling of distinct cell cycle genes. Our analysis revealed that HPV E7 causes deregulation of a large number of cell cycle genes that are normally regulated by DREAM. Deregulation also affects p53 function through disruption of the p53-p21-DREAM pathway (Fig. 3). This mechanism is independent of HPV E6-mediated destruction of p53.

It is widely accepted that pRB controls the G_1_/S checkpoint and that it is required for G_1_/S transition^5^. However, HPV-induced proliferation additionally requires deregulation of the G_2_/M checkpoint. The DREAM complex contributes to the G_2_/M checkpoint through downregulation of cell cycle genes in response to p53 activation^40^. The HPV E7 oncoprotein deregulates target genes of the DREAM complex that comprise G_1_/S and G_2_/M cell cycle genes (Tables 1, 2 and 3 and Figs 1 and 2). Furthermore, the G_1_/S subgroup of DREAM target genes is also bound by pRB-E2F complexes^23^. DREAM binds to these genes either through E2F promoter elements or through a combination of an E2F site and a CHR-like element (CLE)^34^. *E2F1* and *ORC1* are examples for G_1_/S genes that are controlled through an E2F promoter element, while *B-MYB* (*MYBL2*) and *RAD51* are examples for G_1_/S genes controlled through E2F/CLE tandem elements^34^. The p53-mediated downregulation of genes from both groups is abrogated by HPV E7 (Figs 1, 2 and 3). In contrast to G_1_/S genes, the G_2_/M subgroup of DREAM target genes is additionally regulated by MMB-FOXM1 complexes^23^. DREAM binds to these genes either through CHR promoter elements or through CDE/CHR tandem sites^34^. *KIF23* and *Survivin* (*BIRC5*) are examples for G_2_/M genes that are controlled through a CHR element^37, 50, 51^, while *CDC25C* and *PLK4* are examples for G_2_/M genes controlled through CDE/CHR sites^41, 51^, and the p53-mediated downregulation of these genes is abrogated by E7 (Figs 1 and 2). Thus, the data suggest that HPV E7 interferes not only with pRB function but also with DREAM to impair cell cycle checkpoints (Fig. 3).

Except for *RAD51*, all experimentally tested DREAM target genes were correctly predicted by the meta-analysis to be deregulated by HPV E7 (Tables 1, 2 and 3). These observations indicate that threshold settings were so stringent that the computational analysis rather missed candidates than to include false-positive genes. This suggests that the genes in Tables 2 and 3 are indeed high confidence targets deregulated by HPV E7, but that some additional target genes may have been missed. Taken together, our findings provide evidence that DREAM target genes are generally deregulated by HPV E7 expression.

It is important to note that pRB differs in its function from the pRB-like pocket proteins p107 and p130. While all pocket proteins pRB, p107 and p130 bind to LxCxE motifs, only p107 and p130 can be recruited to the MuvB core through an LxSxExL motif in LIN52 to form the DREAM complex^57^. HPV E7 possesses an LxCxE motif through which it binds pocket proteins^8^, and binding of E7 to p107 and p130 inhibits their interaction with the LxSxExL motif in LIN52^57^. Several other viral oncoproteins target the pocket proteins through LxCxE motifs, including adenovirus early-region 1A (E1A) and large T antigens of several polyomaviruses, such as SV40, JCV and BKV^58^. Consistent with this notion, also SV40 large T was reported to impair DREAM function^59, 60^.

It is established that HPV destroys p53 function through marking it for degradation by the E6 oncoprotein^10, 11^. This mechanism may be sufficient to block p53 activity completely. However, p21 is a central effector of the p53 response, and p21 can be activated independently of p53, for example through the MAPK and TGFβ pathways^61^. Also in the absence of HPV E6, we observe that the p53-p21-DREAM pathway is intercepted further downstream by E7 interfering with DREAM function and host cell cycle arrest (Tables 1, 2 and 3 and Figs 1 and 2). The data indicate that HPV employs several means to disrupt cell cycle checkpoints (Fig. 3).

In summary, the data reveal that deregulation of DREAM function by the HPV E7 oncoprotein may contribute substantially to the development of the many cancer types caused by HPV.

## Electronic supplementary material

Supplementary Figure S1

Supplementary Table S1
