# Supplementary figures and images for: Human papilloma virus E7 oncoprotein abrogates the p53-p21-DREAM pathway
Source: Sci Rep. 2017 Jun 1;7:2603. doi: 10.1038/s41598-017-02831-9 (PMC5453983; doi:10.1038/s41598-017-02831-9)

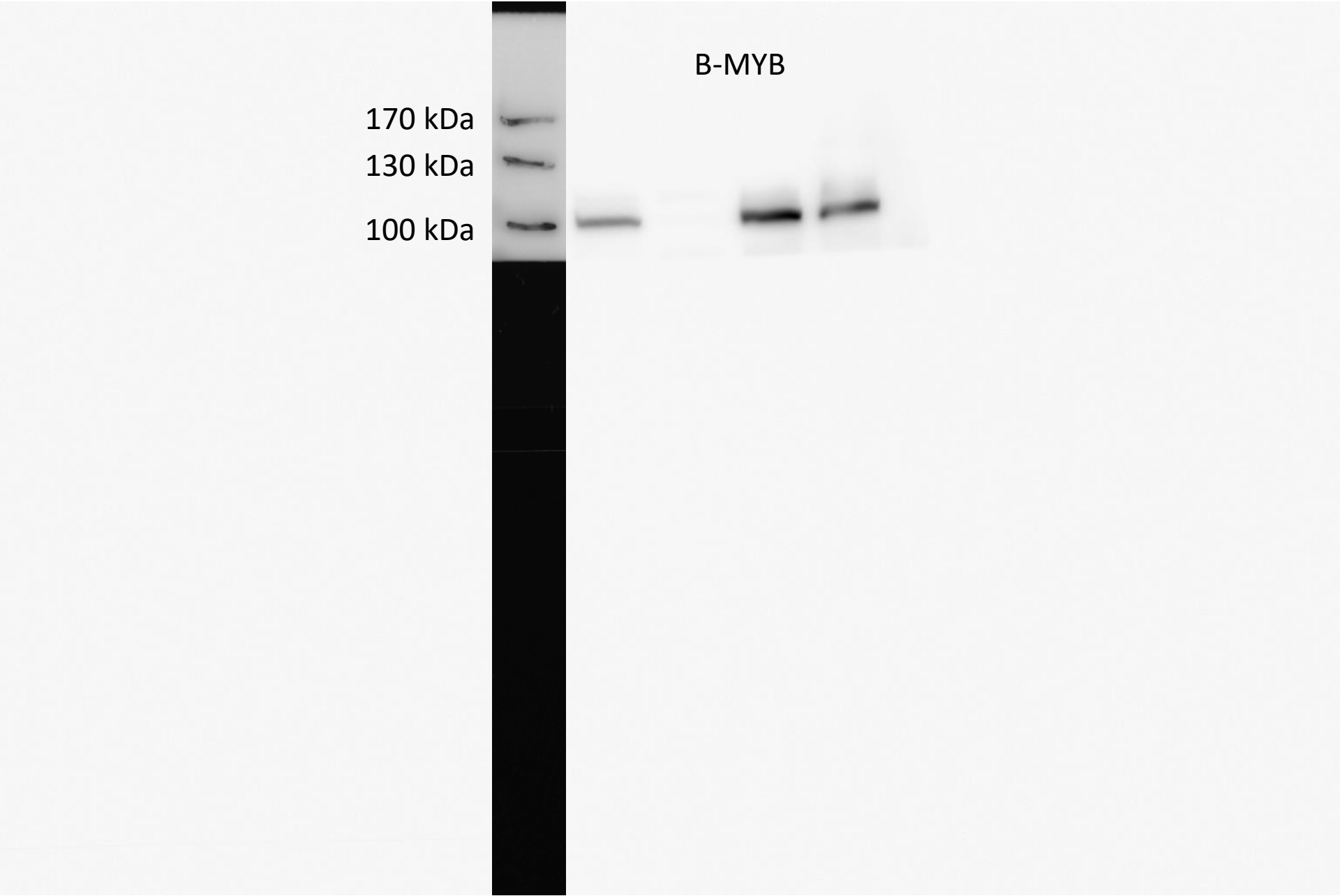

70 kDa

55 kDa

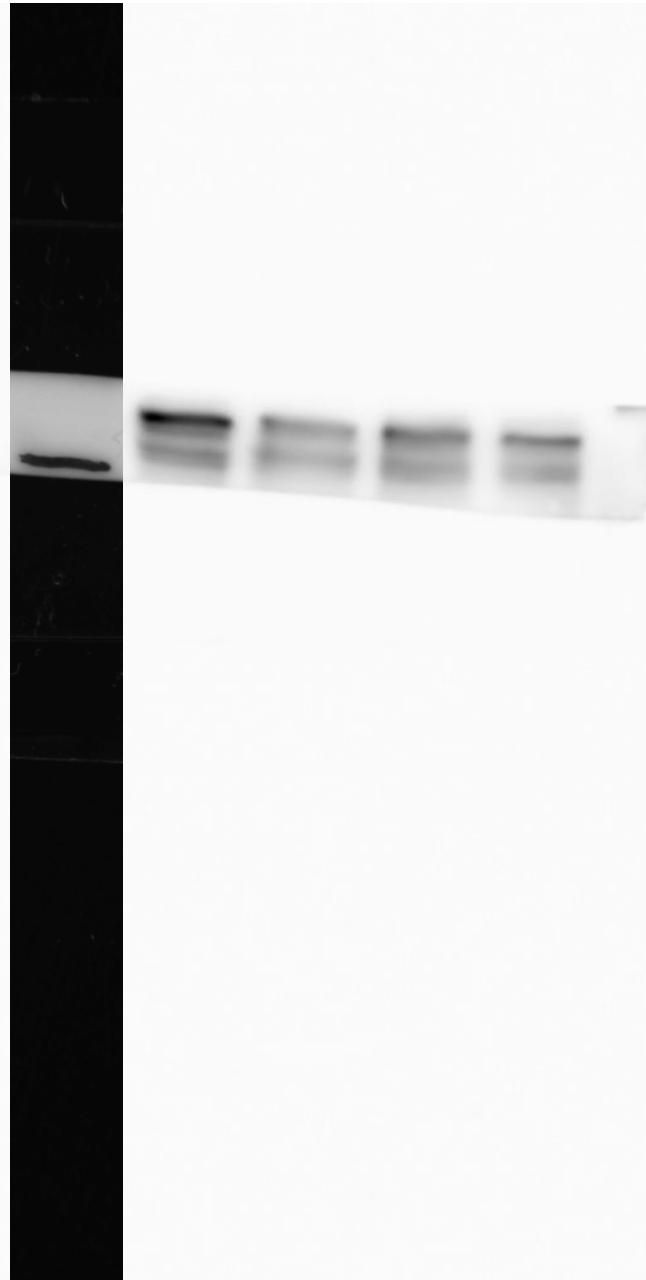

E2F1

170 kDa

130 kDa

100 kDa

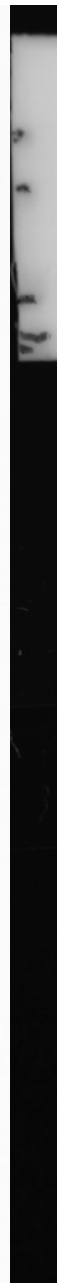

KIF23

70 kDa

55 kDa

40 kDa

35 kDa

25 kDa

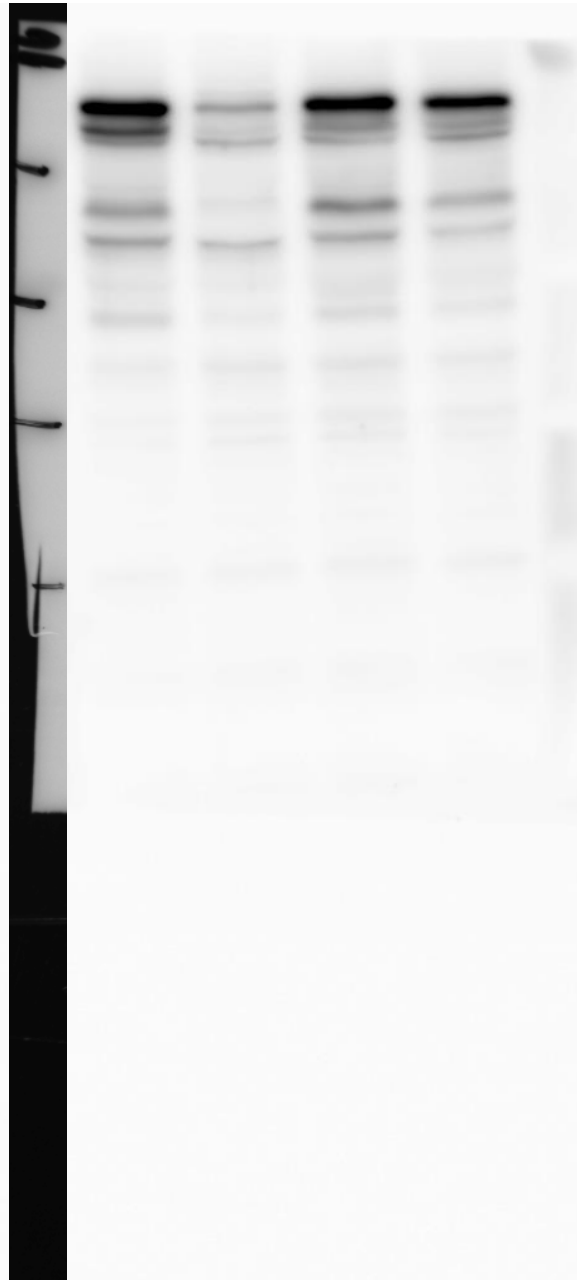

CDC25C

40 kDa

35 kDa

$\beta$ -actin

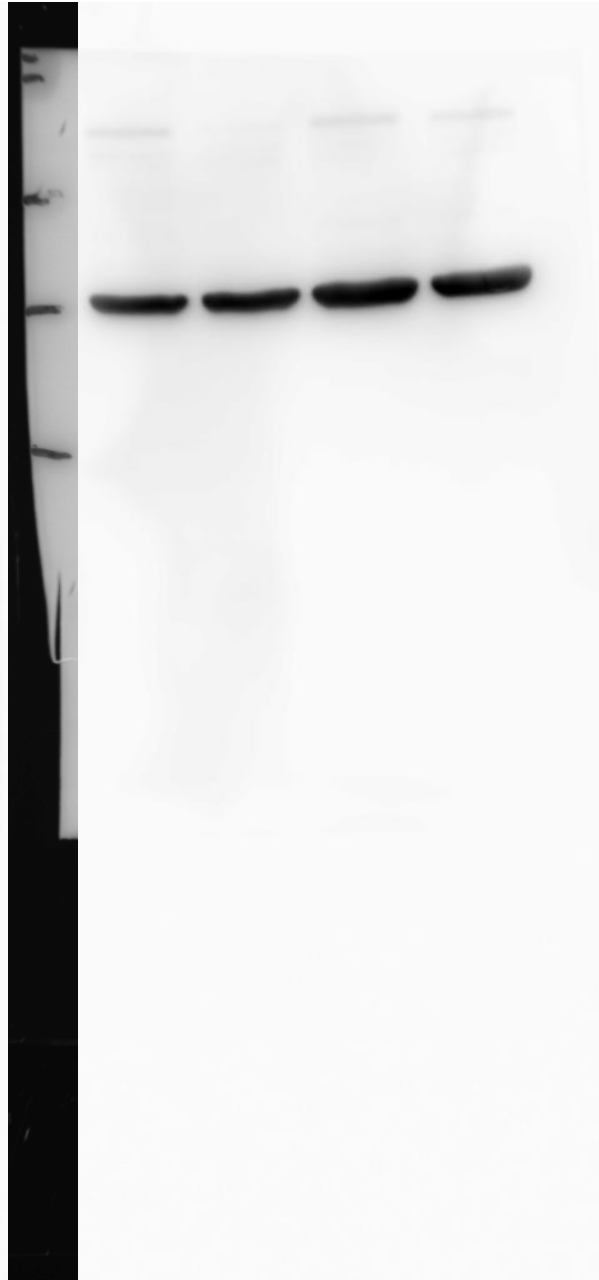

Supplement: Supplementary file 1 — Supplementary Figure S1 [file 41598_2017_2831_MOESM1_ESM.pdf]
